# Mercury dynamics in macroinvertebrates in relation to environmental factors in a highly impacted tropical estuary: Buenaventura Bay, Colombian Pacific

**DOI:** 10.1007/s11356-019-06970-6

**Published:** 2019-12-10

**Authors:** Diego Esteban Gamboa-García, Guillermo Duque, Pilar Cogua, José Luis Marrugo-Negrete

**Affiliations:** 1 Facultad de Ciencias Agropecuarias, Universidad Nacional de Colombia, Palmira, Colombia; 2 Facultad de Ingeniería y Administración, Universidad Nacional de Colombia, Palmira, Colombia; 3 Facultad de Ciencias Básicas, Universidad Santiago de Cali, Cali, Colombia; 4 Facultad de Ciencias Básicas, Universidad de Córdoba, Montería, Colombia

**Keywords:** Epibenthic invertebrates, Tropical estuary, Human health, Artisanal fisheries, Macroinvertebrates

## Abstract

The environmental health of Buenaventura Bay, a highly impacted tropical estuary, is influenced by numerous human activities, including mining upstream. Large- and small-scale fishing plays an important role in the local economy, so we investigated the dynamic processes of bioaccumulation of mercury at basal trophic levels. Four samples were taken at each of the four locations in Buenaventura Bay during each of the four seasons of 2015. We measured the total mercury content (T-Hg, dry weight) in sediments and in muscle tissue across 17 macroinvertebrate species. The most abundant were the blue crab (*C. arcuatus*) and the mantis shrimp (*S. aculeata aculeata*). Blue crab showed an average muscle T-Hg value ​​exceeding the limit of 0.2 g·g^-1^, which is the maximum T-Hg level suggested for food consumption by vulnerable humans and populations: pregnant women, children, and the community that feeds from this source of protein on a daily basis. It was found that, 6.22% of individuals exceeded the 0.5 g·g^-1^ level, which is the maximum T-Hg level suggested for food consumption by the general population: the population that consumes it sporadically. Significantly high values ​​of T-Hg in blue crab and mantis shrimp occurred during low salinity conditions in the estuary, suggesting that Hg mainly originates from river runoff during the rainy season. Nevertheless, the biota-sediment accumulation factor (BSAF) was favored in high salinity, which could mean greater availability of Hg for higher levels of the estuarine food web in the dry season and in marine waters. In general, the T-Hg levels in some samples exceeded 0.2 g·g^−1^. Therefore this pollutant must be monitored due to its biomagnification potential and as a threat to human health, especially that for the local population of fishermen and their families.

## Introduction

Coastal ecosystems are recognized for their wide variety of habitats that permit a broad diversity of species and generate high biological productivity. At the global level, marine resources provide at least 15% of the animal protein for 2.9 billion people and livelihoods for 520 million people (FAO 2009). Of this global productivity, 5.2% comes from estuaries, which sustain commercially important artisanal and coastal fisheries (Blaber 2013; Houde and Rutherford 1993). Nevertheless, these areas are susceptible to the incursion of pollutants affecting natural dynamics. Particularly intense in these environments is the retention of heavy metals (Bayen 2012), which may have both socioeconomic and ecological implications.

Mercury (Hg) contamination of marine organisms has socioeconomic implications, as high concentrations of the metal in these organisms’ muscle tissue would potentially violate regulations for human consumption (Costa et al. 2016; Padula et al. 2016), thereby affecting fish trade. In the highly impacted coastal estuary of Buenaventura Bay, Colombia, artisanal fisheries account for 50% of total fish production, and about 3000 inhabitants depend heavily on this activity for their income and food supply (Escobar Cárdenas 2009). On the Pacific coast of Colombia, the annual per capita fish consumption is 250 kg, compared to the national average of 4.5 kg (Villanueva and Flores-Nava 2019), highlighting the relevance of the human health concern.

Although Hg can occur naturally in estuaries through atmospheric transport and deposition or through river discharge, the main sources globally are chlor-alkali plant discharge, fossil fuel burning, dental waste, and gold mining (Amos et al. 2014; Costa et al. 2012; Horowitz et al. 2014). On the Pacific coast of Colombia, Hg mainly originates from the mining of alluvial gold, whose polluting waste is transported by the rivers that flow into the coastal zones (CVC 2010), reaching even estuaries that are protected areas (Duque et al. 2018)

In addition, the presence of Hg in sediments has been assigned a moderate ecological risk level (Cardoso et al. 2009; Guo et al. 2010; Li et al. 2019). Hg hosted in sediments can be accumulated in the long term by organisms, harming their reproductive and developmental processes (Hong et al. 2012). This issue, along with other stressors, can lead to a decline in populations and even present a threat to the dynamics of estuarine communities throughout the trophic web (Boening 2000; Lopes et al. 2014). The socioeconomic and ecological implications are important to address, as in some estuarine ecosystems, Hg concentrations have increased in both organisms and environmental compartments (Bayen 2012; Lamborg et al. 2014), raising the risk to human health.

In particular, in Buenaventura Bay, the presence and accumulation of Hg have been reported in sediments and organisms (Duque and Cogua 2016; Gamboa-García 2017; (Gamboa-García et al. 2018; Panesso Guevara 2017; Velásquez and Cortés 1997) and even in human blood and hair (Ardila Benavides 2000). This bioaccumulation occurs because Hg is not biodegradable and accumulates in biotic and abiotic compartments. Organic Hg (e.g., methyl mercury, MeHg) is stored not only in fatty tissue but also in the muscle. In the blue crab (*Callinectes sapidus*), 98%–100% of the total mercury (T-Hg) occurs as methyl mercury (Adams and Engel 2014), so in the present study, only T-Hg was measured in the muscle of macroinvertebrates. According to Adams and Engel (2014), the principal route of entry of T-Hg is by feeding; thus, T-Hg levels in the muscle serve as evidence of bioaccumulation. Moreover, due to the biomagnification processes, Hg can be transferred via the food web to human consumers (Olivero-Verbel et al. 2002; Taylor et al. 2014). Vulnerable groups, such as pregnant women and fishermen who live along coastal areas, can be at particular risk from direct or indirect exposure to Hg (Ausili et al. 2008; Costa et al. 2012; Zhang and Wong 2007).

On the Pacific coast of Colombia, the average per capita fish consumption is about 2.68 kg per week, while the average per capita macroinvertebrate consumption, especially of mollusks such as *Anadara* sp., is around 0.8 kg per week. Thus, by weight, macroinvertebrates account for about 23% of the total consumption of marine fish and shellfish. Therefore, between 4% and 38% of the Hg content in both blood and hair of local fishermen has been assigned to macroinvertebrate intake (Ardila Benavides 2000). Due to the socioeconomic and ecological implications of Hg contamination in estuaries, this study mainly aims to determine the bioaccumulation of T-Hg of epibenthic macroinvertebrates and the mercury dynamics in the sediments related to key environmental variables.

## Materials and methods

### Study site

**Fig. 1:**
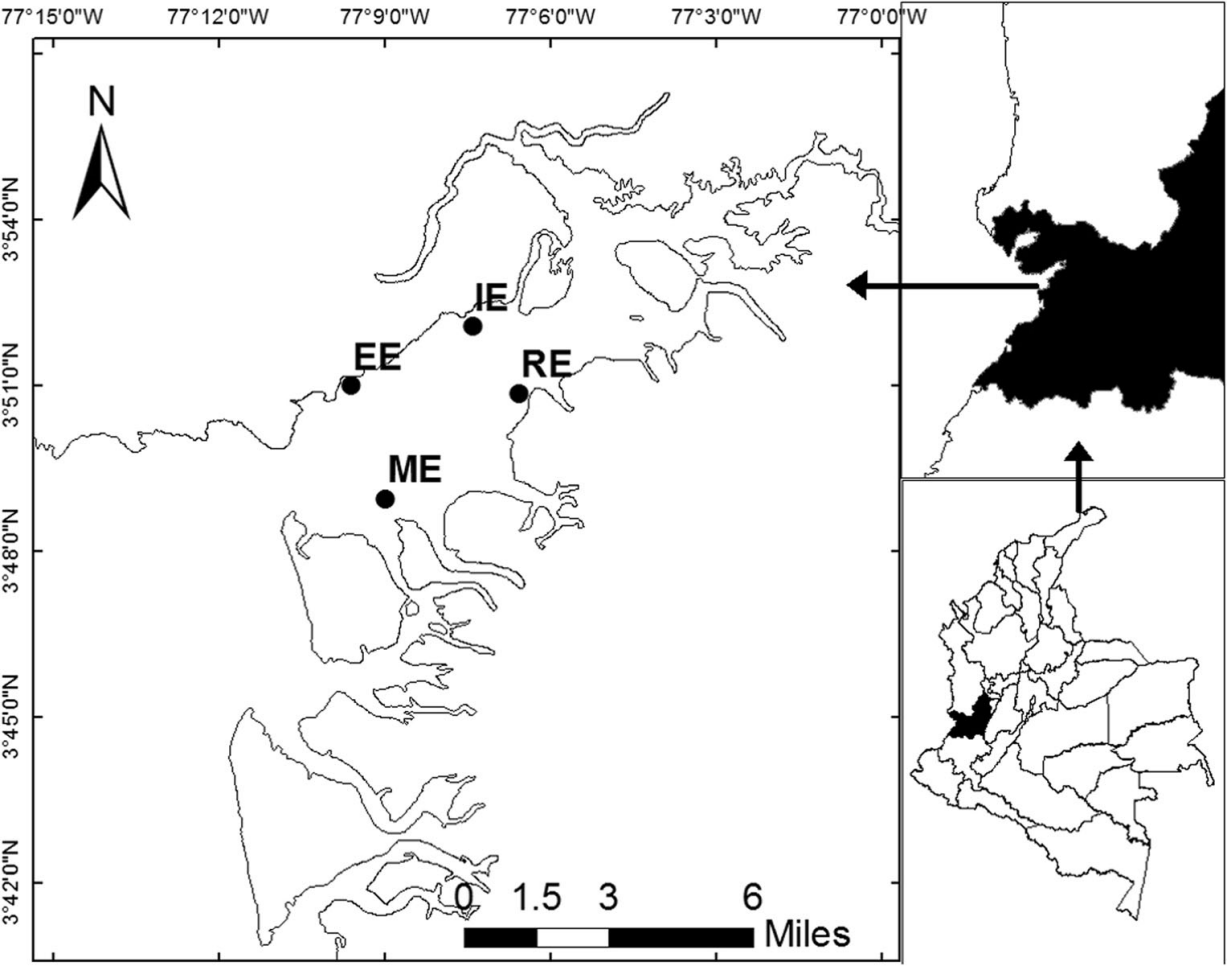
Study area: Buenaventura Bay. *RE* River Estuary; *IE* Internal Estuary; *EE* External Estuary; *ME* Marine Estuary. Source: Adapted from SIGOT

This study was conducted in Buenaventura Bay (3° 44′–3° 56′ N, 77° 01′–77° 20′ W). The bay’s width ranges from 3.4 km at the ocean outlet to 5.5 km within. It is approximately 30 km long, with a narrow and elongated shape (Fig. 1). Its average depth is 5 m, without appreciable variability, with only the central channel achieving a depth greater than 15 m (Otero 2005). The tide is semidiurnal, with an average daily range of 3.7 m, and the water temperature ranges from 25.7 to 29.8 °C (Cantera et al. 1995; Otero 2005).

This estuary contains two areas: the inner bay and outer bay. This study focused on the outer bay, which is characterized by well-mixed, vertically homogeneous water, with a surface-to-bottom salinity difference of less than 2 PSU, at both low and high tides (Otero 2005).

### Sediment, water, and macroinvertebrate sampling

Four samples were taken in each climatic season of 2015: April for dry, June for dry intermediate, September for rainy intermediate, and November for rainy. The sampling was conducted at four stations distributed in Buenaventura Bay according to a salinity gradient. The River Estuary (RE) station, located at 3° 50′ 51.5″ N, 77° 6′ 33.1″ W, was internal and influenced by the discharge of the Dagua River. The Internal Estuary (IE) station, located at 3° 52′ 4.4″ N, 77° 7′ 24.9″ W, was internal and bounded by a rocky coast. The External Estuary (EE) station, located at 3° 50′ 58.7″ N, 77° 9′ 35.9″ W, was external and bounded by a rocky coast. Finally, the Marine Estuary (ME) station, located at 3° 48′ 56.5″ N, 77° 8′ 58.4″ W, was external and influenced by the discharge of the Anchicayá River. The stations were mutually separated by an average distance of 4 km (Fig. 1).

For each season and station, water samples were taken for physicochemical variables, sediments for granulometry, and macroinvertebrates for community structure and abundance. Each sample included three replicates, for a total of 48 samples. To determine the composition of the sediment samples, a full 5 vertical cm of the substrate was collected through a PVC core, which was 4.5 cm in diameter and 20 cm in length. For the surface water samples, a Thermo Scientific portable multimeter was used to determine the physicochemical variables: temperature (°C), salinity (SPU), pH, and dissolved oxygen (OD mg·L^−1^). In addition, transparency was measured with a Secchi disk, depth was measured using a depth sounder floating-depth gauge, and geographical coordinates were measured with GPS (Garmin).

Epibenthic macroinvertebrates were sampled using a trawl boat with a 2.54-cm mesh and a working width of 8 m. Independent trawls were developed every 10 min, with each experimental unit including three replicates for a total of 48 trawls. The organisms and sediments were stored in plastic bags, refrigerated, and then taken to the laboratory to be stored at −20 °C.

### Sediment and macroinvertebrate analysis

From each core sample of sediments and its replicates, the top 5 cm were extracted and composited into a single sample. Five grams of this sample was used for organic matter content measurement by ignition (Danovaro 2010), and 30–40 g was used for granulometry, through a mechanical treatment using sieves for particles ranging from 50 to 1000 μm.

Macroinvertebrates were counted and identified. Sex and reproductive status were determined, when was possible, and weight and height were measured. The specimens were classified taxonomically at the species level, using FAO taxonomic codes (Fischer et al. 1995), internet databases (WoRMS: World Register of Marine Species), and literature review (Baltazar 1997; Cardoso and Hochberg 2013; Lazarus-Agudelo and Cantera-Kintz 2007; Lemaitre and Alvarez León 1992; Neira and Cantera 2005; Pineda and Madrid 1993).

### Hg quantification in sediments and organisms

The determination of total Hg in sediments and macroinvertebrate muscle was performed as follows. For each of the 48 sediment samples, 50 mg of each sample was extracted. For each macroinvertebrate sampled, one sample was taken from either the chela muscle for crabs (Olivero-Verbel et al. 2008), adductor muscle for bivalves, mantle for squid, or abdomen for shrimp; these structures and others for the rest of the species corresponded to the main muscle of each organism. Hg measurements were performed in the macroinvertebrate muscle, because in this tissue, the MeHg proportion of T-Hg has been reported to be approximately 98–100% (Adams and Engel 2014), and the MeHg is evidence of the entry of mercury into organisms through food. Therefore the muscle Hg concentrations are most likely to help identify the diet as the route of entry of Hg to the organisms and to show clear evidence of bioaccumulation (Taylor and Calabrese 2018).

For each of the 311 samples through the different seasons and stations, 5–10 mg of the muscle was extracted. Procedures were executed using plastic and ceramic instruments, to avoid contamination by metals. For the total Hg analysis, the direct measurement method was used, following USEPA (2007). Freeze-dried samples were analyzed by thermal decomposition and atomic absorption spectrometry with gold amalgamation using a Milestone DMA-80 Direct Mercury Analyzer (Milestone GmbH, Germany), with a limit detection of 0.2 ng. For quality control, a solution was prepared, and two samples of 50-μL solution were extracted, each with 50 ng of Hg. A recovery rate of 96 ± 2% was obtained. In addition, the DORM2-certified standard 4.64 ± 0.26 μg·g^-1^ was used, where the DMA-80 reading showed variability of less than 6% (USEPA 2007).

## Data analysis

### Physicochemical variation and its influence on T-Hg content in sediments

The measured physicochemical variables of water included salinity, pH, oxygen concentration (CO, mg·L^−1^), and transparency (Tr, cm). The measured sediment composition variables included the proportions of organic matter (%MO), gravel and very coarse sand (%G), coarse sand (%CS), medium sand (%MS), fine sand (%FS), silt (%Si), and clay (%A). For each of these variables and for T-Hg content in sediments, normality of the distribution was reviewed, and residual graphs were examined to confirm normality and homogeneity of variance. Where necessary, an appropriate transformation was performed to better approximate normality (Green 1979; Mead 2017).

The number of environmental variables was reduced using principal component analysis (PCA) based on an array of correlations (Chatfield and Collins 2018; Farcomeni and Greco 2016). PCA was performed using the Princomp procedure in SAS 9.4 (SAS 2002), to reduce the 13 environmental variables to 8 uncorrelated variables that explained 65.3% of the variance in the experiment.

Significant differences (*p* < 0.05) between environmental variables (i.e., physicochemical parameters of the water and granulometry) were examined using a two-way multivariate analysis of variance (MANOVA), with climatic season and location as main factors, using the general linear model and the mean-squared deviation criterion, in SAS 9.4 (SAS 2002). To improve interpretation, ANOVA was performed to test Type III error. Finally, Tukey’s honest significance test was used to identify the climatic season or location in which the variable in question presented significant differences.

To evaluate the differences in T-Hg content among the sediment samples, a two-way ANOVA was performed, with location and climatic season as the main factors, using the general linear model and the least square means criterion, in SAS 9.4 (SAS 2002). Type III error was examined to improve interpretation, and Tukey’s honest significance test was used to identify the climatic season or location in which the T-Hg content in the sediments presented significant differences.

A stepwise multiple regression analysis was developed to determine the physicochemical variables related to T-Hg content in sediments (Ghani and Ahmad 2010; Mead 2017). Collinearity among independent variables was evaluated by examining the variance inflation factors (VIFs). In this evaluation, variables were considered independent if VIF values ​​were close to 1 and collinear when the value reached 10 or above (Allison 2012; Farina et al. 2018; O’brien 2007). A value of *p* < 0.05 was chosen as the input and output values to identify the set of variables that were important in the description of the dependent variable. The highest value of F was used at each step to identify the variable that contributed the most to the *R*^*2*^ value. Subsequent variables were chosen similarly; however, after each new addition, all variables were examined to ensure that they still met the model criterion (*p* < 0.05). If the variable was not significant, it was eliminated from the model.

### T-Hg content variation in macroinvertebrate muscle

The most abundant species present at the four locations during the four seasons were selected for the analyses of covariance. Normality tests were conducted for the size, weight, and concentration of T-Hg in the muscle. Normalization adjustment to each distribution was undertaken with potential Box-Cox transformations (Statgraphics Centurion XVI 2009). After the transformation, the residuals and residual graphs were examined to evaluate the assumption of normality and homogeneity of the variance. A simple correlation was performed between the size and weight of macroinvertebrates in order to work with just one independent covariable. As size affects the accumulation of Hg in organisms (Andersen and Depledge 1997; Coelho et al. 2013), it is an important dimension to be captured as a covariable. The relation between T-Hg content in the muscle and macroinvertebrate size was evaluated using linear regression. Where the relation between T-Hg and size was significant, covariance analysis (ANCOVA) was used for comparisons among locations and among seasons (Verdouw et al. 2011). For the ANCOVA model, the general linear model and Tukey’s test were used in SAS 9.4 (SAS 2002).

### Influence of sediment T-Hg concentration and physicochemical variables on T-Hg content in macroinvertebrate muscle

A univariate multiple regression analysis was performed to determine which physicochemical variables of the water and sediment, including sediment T-Hg content, influenced the macroinvertebrate muscle’s T-Hg content. Collinearity between the independent variables was evaluated by examining the VIFs (Allison 2012; Farina et al. 2018; O’brien 2007). The same criteria as those used in the univariate multiple regression model described above were used here.

### Variation among macroinvertebrates in the biota-sediment accumulation factor of T-Hg

To establish the empirical reality of bioaccumulation, a regression among specimens of the most abundant species was performed between muscle T-Hg concentration and size (Reichmuth et al. 2010). To assess the bioaccumulation process, the biota-sediment accumulation factor (BSAF) was calculated for all taxa collected: BSAF = T-Hg concentration in muscle of biota/T-Hg concentration in sediments (Taylor and Calabrese 2018). ANCOVA was performed with macroinvertebrate size as a covariable to determine BSAF differences among the locations and seasons. For the ANCOVA model, the general linear model and Tukey’s test were used in SAS 9.4 (SAS 2002). Although other routes of Hg entry to the organisms (concentration via gills and water consumption) could influence the BSAF measurements, BASFs were calculated site-specifically, so that neither inter-location nor inter-seasonal variability would not be masked by overall trends in metal BSAF.

## Results

### Physicochemical variation and its influence on sediment T-Hg content

**Fig. 2:**
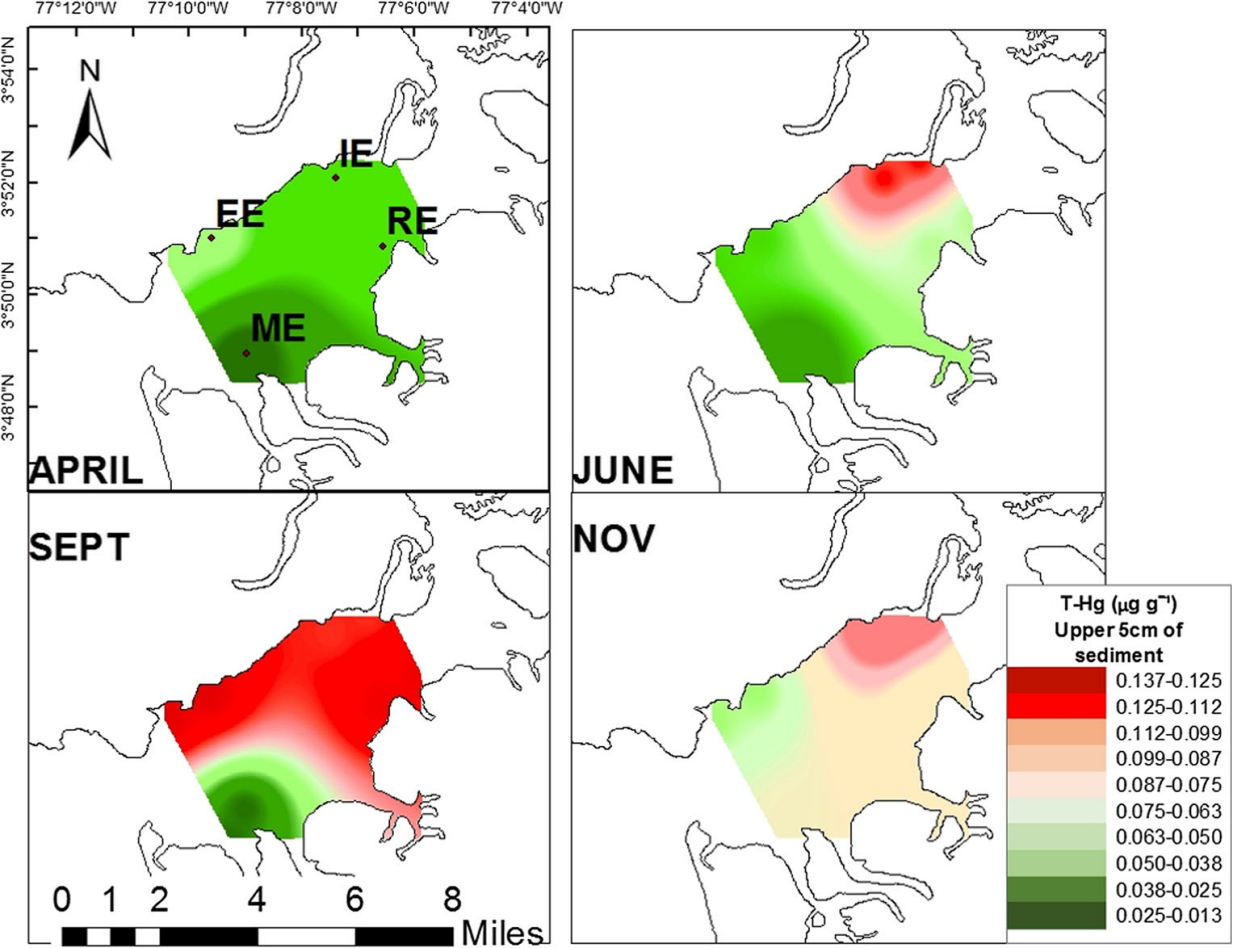
T-Hg gradient map in the upper 5 cm of sediments (μg·g^-1^ dw) by season and location

In Buenaventura Bay, the sediment T-Hg concentration varied between seasons from an average of 0.040 to an average of 0.098 μg·g^-1^ (dry weight = dw) and between locations from an average of 0.032 to an average of 0.096 μg·g^-1^ dw. The months of highest concentration of T-Hg in sediments were June, September, and November, while the stations with the highest concentrations were RE and IE. The ME station, closest to open-ocean conditions, had the lowest sediment T-Hg values, except for November, when an increase was observed (Fig. 2). September and November showed the highest sediment organic matter values and the highest percentages of the coarse fraction of the sediment (gravel + coarse sand). These months also showed the lowest values for transparency, pH, and salinity. Internal stations (RE and IE) showed higher percentages of gravels, silt, as well as greater depth, while fine sand %, dissolved oxygen, pH, and salinity showed lower values at RE and IE as compared to that at other stations.

The percentage of organic matter was a good predictor of the sediment T-Hg content (*R*^*2*^ = 0.74, *p* < 0.0001). The content of T-Hg in sediments collected in 2015 tended to be higher in seasons and locations where the sediment contained a higher % organic matter (%MO). There were significant differences by season (ANOVA, *F* = 26.15, *p* < 0.0001), location (ANOVA, *F* = 45.51, *p* < 0.0001), and season–location interaction (ANOVA, *F* = 12.22, *p* < 0.0001).

In Buenaventura Bay, the months with the highest sediment T-Hg concentrations were September (0.098 ± 0.016 μg·g^-1^ dw, confidence = 95%) and November (0.08 ± 0.006 μg·g^−1^ dw, confidence = 95%). The season with the lowest concentration was April (0.04 ± 0.005 μg·g^−1^ dw, confidence = 95%, Tukey, *p* < 0.05). The location with the highest sediment T-Hg content was IE (0.096 ± 0.011 μg·g^-1^ dw, confidence = 95%), followed by RE (0.079 ± 0.01 μg·g^−1^ dw, confidence = 95%) and EE (0.073 ± 0.012 μg·g^−1^ dw, confidence = 95%), with the lowest sediment T-Hg content at ME (0.032 ± 0.008 μg·g^−1^ dw, confidence = 95%, Tukey, *p* < 0.05). In general, for most stations, the lowest sediment T-Hg concentrations occurred during periods of marine conditions (April and June). However, June (dry intermediate season) at the internal station IE showed an increase (Tukey, *p* < 0.05). The highest sediment T-Hg values occurred in the seasons of lower salinity (September and November) for most stations, except for the decrease in September at the external station ME (Tukey, *p* < 0.05).

In the stepwise multiple regression analysis, the composition of the sediment was a better predictor of sediment T-Hg content (*R*^*2*^ = 0.90, *p* = 0.001) as compared to the physicochemical variables of water (*R*^*2*^ = 0.40, *p* = 0.002). In particular, sediment T-Hg content was higher when organic matter, clay, gravel, very coarse sand, and silt were higher. With respect to the physicochemical conditions of water, sediment T-Hg content was higher at greater water depths but lower when pH, temperature, and transparency were higher. Ten physicochemical variables were used to predict sediment T-Hg content (Table 1), including only those variables that were not collinear. All models were significant (*p* ≤ 0.05), and the most notable variables showed significant correlations.

**Table 1:** Multiple regression analysis of sediment total Hg content in relation to environmental variables of sediment and water. Variables are reported in the order in which they entered the model, that is, in decreasing order by *F* value (*p* ≤ 0.05). The sign of the relation between T-Hg concentration and each environmental variable is represented by sign, and the Pearson product–moment correlation coefficients are provided in parentheses. The level of significance to retain the variables in each model was *p* ≤ 0.05, except for the variables in italics, for which 0.05 ≤ *p* ≤ 0.15. Strong Pearson correlations are indicated in bold. All distributions Box-Cox transformed.

	***R***^***2***^	**Variable 1**	**Variable 2**	**Variable 3**	**Variable 4**	**Valor de F**	***p*>*F***
**Sediment variables**							
** Total Hg content in sediments**	0.90	**(+)MO(0.90)**	**(+)Clay(0.85)**	(+)Gravels(0.60)	(+)Silt(0.40)	51.9	<0.001
**Water variables**							
** Total Hg content in sediments**	0.40	*(−)pH(0.30)*	(−) Transparency(0.41)	(+)Depth(0.32)	*(−)* *Temperature(0.16)*	5.6	0.002

Significant Pearson’s correlations with sediment T-Hg content were found for percentage of organic matter (*p* = 0.001), percentage of gravel and very coarse sand (*p* = 0.02), and percentage of clay (*p* = 0.0001). According to Pearson’s correlations, organic matter, gravels, and very coarse sands and clays explained, respectively, 90%, 60%, and 85% of T-Hg content variance in sediments.

### Variation in T-Hg content in macroinvertebrate muscle

The measurement of total Hg in the macroinvertebrate muscle in Buenaventura Bay in 2015 showed that all species contained Hg in their tissues. The individual with the highest concentration of T-Hg in all seasons and locations was a mature male of *Callinectes arcuatus* with 0.75 μg·g^−1^ dw, while individuals showing lower metal concentrations included the freshwater shrimp *Macrobrachium tenellum* (0.019 μg·g^−1^ dw) and the sea star *Luidia columbia* (0.024 μg·g^−1^ dw) (Table 2). The species that showed the highest T-Hg concentrations were the crabs *Callinectes arcuatus* and *Callinectes toxotes.*

**Table 2:** Means and standard deviations of T-Hg content (μg·g^−1^, dry weight) in macroinvertebrate muscle, where 0.2 μg·g^−1^ is the maximum T-Hg level suggested for food consumption by this vulnerable human population. The vulnerable population in this case refers to pregnant women, children, and the community that feeds from this source of protein on a daily basis; the general population is the population that consumes it sporadically. The maximum T-Hg level suggested for food consumption by the general population is 0.5 μg·g^−1^ (0.5 ppm).

					**% of samples rather than**	
**(Sub)species**	**n**	**Mean [T-Hg] μg g**^**−1**^	**SD [T-Hg] μg g**^**−1**^	**range [T-Hg] μg g**^**−1**^	**0.2 μg g**^**−1**^	**0.5 μg g**^**−1**^
*Callinectes toxotes*	4	0.273	0.059	0.359–0.234	100	
*Menippe obtusa*	1	0.255			100	
*Callinectes arcuatus*	209	0.175	0.144	0.751–0.03	24.4	6.22
*Lolliguncula panamensis*	17	0.086	0.022	0.13–0.053		
*Squilla aculeata aculeata*	33	0.084	0.036	0.196–0.045		
*Rimapenaeus byrdi*	9	0.075	0.032	0.143–0.035		
*Penaeus stylirostris*	1	0.07				
*Anadara reinharti*	1	0.067				
*Penaeus brevisuturae*	2	0.06	0.012	0.069–0.052		
*Penaeus californiensis*	5	0.058	0.024	0.08–0.03		
*Penaeus occidentalis*	15	0.056	0.021	0.114–0.035		
*Clibanarius lineatus*	3	0.055	0.019	0.076–0.04		
*Penaeus vannamei*	4	0.05	0.008	0.058–0.041		
*Penaeus pacificus*	3	0.049	0.022	0.071–0.028		
*Polymesoda inflata*	1	0.045				
*Macrobrachium tenellum*	1	0.02				
*Luidia columbia*	2	0.012	0.017	0.024–0		

For the year 2015, all individuals of the species *C. toxotes* in Buenaventura Bay showed muscle T-Hg levels higher than 0.2 μg·g^−1^ dw, which is the maximum T-Hg level suggested for food consumption by this vulnerable human population. The vulnerable population in this case refers to pregnant women, children, and the community that feeds from this source of protein on a daily basis; the general population is the population that consumes it sporadically. The crab *C. arcuatus* was the only species of macroinvertebrate whose individuals showed muscle T-Hg levels exceeding 0.5 μg·g^−1^ dw, which is the maximum T-Hg level suggested for food consumption by the general population (μg·g^−1^, WHO 1990) (Table 2).

The species *C. arcuatus* was collected in all seasons and locations abundantly, so the analysis of covariance was performed using this species. First, differences by sex were examined in the muscle T-Hg content of *C. arcuatus* (ANOVA, *p* = 0.13), then by reproductive status (ANOVA, *p* = 0.0001), and then in the interaction of these two variables (ANOVA, *p* = 0.53). A significant relation was found between muscle T-Hg concentration and crab size (R^2^ = 0.43, *p* < 0.0001); therefore, ANCOVA was undertaken, with the width of the crab carapace (WC) as a covariate. WC was found to have a significant effect on the measured Hg levels in *C. arcuatus* from all seasons (ANCOVA, *p* = 0.0002) and locations (ANCOVA, *p* = 0.0336).

The crab muscle’s T-Hg content data were standardized by size in order to accurately evaluate the seasonal and zonal differences. The season of highest size-standardized crab muscle T-Hg concentration was November (Tukey; November vs. April, *p* < 0.0001; November vs. June, *p* < 0.0001; November vs. September, *p* < 0.05). The IE location showed the highest crab muscle T-Hg concentration, with the lowest occurring at ME. Throughout the seasons and locations, November was the month of the highest crab muscle T-Hg concentration (Table 3).

**Table 3:** Size-standardized average crab muscle T-Hg content of *C. arcuatus* by season and location, estimated by least squares (±SD). The letters read vertically indicate significant differences (Tukey) with a significant two-way interaction (*p* ≤ 0.05). Each average was calculated from a pool of samples for a total of 209 samples. RE = River Estuary, IE = Inner Estuary, EE = External Estuary, ME = Marine Estuary.

**Month**	**Location**	**Standardized content of T-Hg (μg g**^**−1**^**mm**^**−1**^**)**
**April**	**EE**	0.013 ± 0.004 C
	**ME**	0.014 ± 0.003 C
**June**	**EE**	0.019 ± 0.006 C
	**IE**	0.020 ± 0.003 C
	**ME**	0.018 ± 0.005 C
	**RE**	0.019 ± 0.015 C
**September**	**EE**	0.022 ± 0.013 BC
	**IE**	0.025 ± 0.019 BC
	**ME**	0.015 ± 0.005 C
**November**	**EE**	0.029 ± 0.014 AB
	**IE**	0.037 ± 0.016 A
	**ME**	0.033 ± 0.021 A
	**RE**	0.032 ± 0.016 A

For the species *C. arcuatus*, the variance in crab muscle T-Hg content was explained nearly equally by the physicochemical conditions of the water (*R*^*2*^ = 0.23, *F* = 15.2, *p* < 0.0001) and the granulometry of the sediment (*R*^*2*^ = 0.23, *F* = 20.29, *p* < 0.0001). The crab muscle T-Hg content tended to be high when both sediment T-Hg concentration and percentage of coarse sand were high, as well as when the following variables were low: salinity, transparency, temperature, percentage of fine sand, and percentage of silt.

Fifteen environmental variables were used in predicting crab muscle T-Hg content, including in the model only those variables that were not collinear (Table 4). The water and sediment models were both significant (*p* ≤ 0.05), and the most notable variables showed significant correlations with crab muscle T-Hg content. Among water parameters, the following variables were significantly correlated with crab muscle T-Hg content: salinity (*p* < 0.0001), transparency (*p* < 0.0001), sediment T-Hg concentration (*p* < 0.05), and temperature (*p* < 0.05). As for granulometry, the following variables showed significant correlations with crab muscle T-Hg content: percentage of fine sand (*p* < 0.0001), percentage of coarse sand (*p* < 0.001), and percentage of silt (*p* < 0.01). According to the Pearson correlations, the explanation of crab muscle T-Hg content variance by the water parameters was distributed as follows: 39% by salinity, 29% by transparency, 22% by sediment T-Hg concentration, and 14% by temperature. Meanwhile, the granulometric variables that best explained crab muscle T-Hg content variance were the percentage of fine sand (27%), the percentage of coarse sand (23%), and the percentage of silt (19%).

**Table 4:** Multiple regression analysis of total Hg content in macroinvertebrate muscle in relation to environmental parameters of sediment and water. Variables are reported in the order in which they entered the model, that is, in decreasing order by *F* value (*p* ≤ 0.05). The sign of the relation between T-Hg concentration and each environmental variable is represented by sign, and the Pearson product–moment correlation coefficients are provided in parentheses. The level of significance to retain the variables in each model was *p* ≤ 0.05, except for the variables in italics, for which 0.05 ≤ *p* ≤ 0.15. Significant Pearson correlations are indicated in bold.

**[T-Hg] in muscle**	**R**^**2**^	**Variable 1**	**Variable 2**	**Variable 3**	**Variable 4**	**F Value**	***p*value**
**Sediment conditions**							
*S. aculeata aculeata*	0.15	**%Clays(+0.78)**				5.5	0.03
*C. arcuatus*	0.23	**%Fine sand (−0.27)**	**%Sand(+0.23)**	%Silt(−0.19)		20.29	0.0001
**Water conditions**							
*S. aculeata aculeata*	0.47	**pH (−0.76)**	***Dissolved oxygen(−0.65)***			13.19	0.0001
*C. arcuatus*	0.23	**Salinity(−0.39)**	**Transparency(−0.29)**	[T-Hg] in sediments (+0.22)	Temperature(−0.14)	15.2	0.0001

For the mantis shrimp subspecies S*quilla aculeata aculeata,* size (total length, TL) was a good predictor of muscle T-Hg concentration (R^2^ = 0.46, *p* < 0.0001), but there was no significant interaction with the covariate (ANCOVA, TL, and season, *p* = 0.51; TL and location, *p* = 0.35). Therefore, ANOVA was performed, establishing that November was the month of the highest concentration of T-Hg in mantis shrimp muscle (ANOVA, *p* < 0.01, Tukey, November vs. April, *p* = 0.045; November vs. June, *p* = 0.12; November vs. September, *p* = 0.12) (Table 5). There were no significant differences among the stations (ANOVA, *p* = 0.91).

**Table 5:** Average T-Hg content of *Squilla aculeata aculeata* muscle by season and location, estimated by least squares (±SD). The letters read vertically indicate significant differences (Tukey, *p* ≤ 0.05). Each average was calculated from a pool of samples for a total of 33 samples. RE = River Estuary, IE = Inner Estuary, EE = External Estuary, ME = Marine Estuary

**Month**	**Location**	**T-Hg content (μg g**^**−1**^**)**
**April**	**EE**	0.060 ± 0.012 B
	**IE**	0.060 ± 0.019 B
**June**	**ME**	0.068 ± 0.014 AB
	**RE**	0.097 ± 0.010 AB
**September**	**EE**	0.080 ± 0.002 AB
	**RE**	0.062 AB
**November**	**EE**	0.099 ± 0.027 A
	**ME**	0.196 ± 0.043 A
	**RE**	0.118 ± 0.043 A

Using multiple regression analysis, it was determined that for *S. aculeata aculeata*, muscle T-Hg levels were better explained by physicochemical water conditions (*R*^*2*^ = 0.47, *F* = 13.19, *p* = 0.0001) than by sediment granulometric parameters (*R*^*2*^ = 0.15, *F* = 5.5, *p* = 0.03). The T-Hg levels in this shrimp tended to be high when the pH and dissolved oxygen of the water were low and when the percentage of clays in the sediment was high. Ten environmental variables were used in predicting the concentration of T-Hg in the muscle of *S. aculeata aculeata*, including in the model only variables that were not collinear (Table 4). The water and sediment models were both significant (*p* ≤ 0.05), although the best model explained only 47% of the variation. The sediment model explained less than 20% of the variation in shrimp muscle T-Hg levels, and the most notable variables showed significant correlations. The Pearson correlations with shrimp muscle T-Hg content were significant for pH (*p* < 0.005), dissolved oxygen (*p* < 0.05), and % clay (*p* < 0.005). According to the Pearson correlations, pH explained 76% of the variance in shrimp muscle T-Hg content, while dissolved oxygen explained 65%, and the percentage of clay explained 78%.

### Bioaccumulation

For the species *C. arcuatus* in Buenaventura Bay, the analysis of variance of BSAF, having the crab carapace width as a covariate, showed that Hg bioaccumulation varied significantly with body size (*F* = 91.37, *p* < 0.0001), season (*F* = 6.4, *p* < 0.0005), and location (*F* = 9.31, *p* < 0.0001). After reviewing Tukey’s test, it was determined that bioaccumulation was greater in June than in April or September, while bioaccumulation in November was greater than that in September. Regarding the locations, it was established that bioaccumulation was greater at the ME station than that at RE or EE, while bioaccumulation was greater at the IE station than that at RE but lower than that at EE.

## Discussion

### Environmental variation in the study area

During the study in Buenaventura Bay, three representative seasons, as well as three representative zones, were clearly defined: marine, freshwater, and intermediate. Marine conditions were indicated by the occurrence of the highest values ​​of the following variables: salinity, pH, temperature, transparency, dissolved oxygen, percentage of silt, and percentage of fine sand. On the other hand, freshwater conditions were indicated by the lowest values ​​of the same set of physicochemical parameters. This classification is based on studies that have described the physicochemical characteristics of the water and sediments in the bay (Cantera and Blanco 2001; Lucero et al. 2006). According to these studies, the percentage of gravel or coarse sediment was high when the pH, salinity, and transparency were low, indicating the influence of fresh water in the form of discharge from the Dagua and Anchicayá Rivers. Although in the rainy months, currents can drag fine sediments; in this estuary, it was gravel that predominated in the sediments of stations upstream of the mouth of the Dagua River (Lucero et al. 2006).

The marine characteristic season was sampled in June, while the predominantly marine sites were ME and EE. This classification was confirmed by the results of the subsequent Tukey’s test, in which June and the EE and ME locations had the highest values of pH, salinity, transparency, percentage of silt, and percentage of fine sand. In contrast, the period characterized by lower salinity was sampled in November, and the predominantly freshwater stations were RE and IE. The intermediate periods were sampled in April and September, and the intermediate zones were between ME and IE, but they varied with the season. In addition, the season significantly affected the sampling station conditions: during June, marine conditions occurred at all stations, both internal and external, while during November, lower salinity occurred at all stations. In general, the dynamics of environmental conditions of the study area were in line with those reported in other estuaries (de Moura et al. 2012; Nebra et al. 2016) and with estuary paradigms (Elliott and Whitfield 2011).

Our analysis identified the periods of greatest precipitation in the estuary and the zones where the drainage areas of the rivers affected salinity, temperature, dissolved oxygen, and sediment fractions. These processes generated shifts in the physicochemical variables of the estuarine microhabitats, acting as a whole to affect Hg dynamics (Cogua et al. 2012).

### Total Hg (T-Hg) in sediments

The seasons affected by periods of precipitation also showed the highest values of organic matter in the sediment, as did the internal zones, which generally have conditions of lower salinity. This pattern was confirmed by the subsequent Tukey’s test, in which the highest content of organic matter occurred in September. During September and November, freshwater conditions occurred in the estuary, because these months correspond to the rainy season and to the discharge peak of the Dagua (126 m^3^·s^−1^) and Anchicayá (112 m^3^·s^−1^) Rivers. This is the season that shows an increase in the contribution of sediments composed of decomposing organic matter (notably mangroves), as well as in liquid waste emissions from the city of Buenaventura (Cantera and Blanco 2001; Cantera et al. 1995; Lobo-Guerrero 1993).

The distribution of sediment Hg concentration suggests that discharges from the rivers are a source of heavy metal for the estuary, as reported in other studies (Kehrig et al. 2003). On the other hand, it has been determined that bioaccumulation of Hg occurs in organisms in the middle reaches of the Dagua River, due to the presence of metal from mining activities (Hernandez et al. 2013; Torres et al. 2005). These determinations represent another line of argument suggesting the inputs of the Dagua and Anchicayá Rivers as a source of heavy metal for Buenaventura Bay.

In addition, a dilution effect was observed from higher concentration in the internal stations to lower concentration in the external stations. This pattern was confirmed when reviewing Tukey’s test: in the sediments of the IE station, there was a higher concentration of T-Hg, while the strongest dilution occurred at ME. This gradient has also been reported in other estuaries (Cogua et al. 2012; Meng et al. 2014; Shoham-Frider et al. 2007). Taken together, the sediment Hg dynamics suggested an anthropogenic origin of Hg, which would enter the estuary through the rivers and runoff during the rainy season.

Note that during the study in Buenaventura Bay in the months of June, September, and November, Hg concentrations in excess of 0.1 μg·g^−1^ occurred, with 0.1 μg·g^−1^ being the maximum level suggested for human health (WHO 1990). The Hg uptake of estuarine organisms can result from the direct uptake from the dissolved or sedimentary phase (Coelho et al. 2008). It has been reported that the sediment Hg content influences bioaccumulation by benthic organisms (Chen et al. 2009).

As for Hg content and sedimentary organic matter, in this study, it was determined that the highest Hg concentrations occurred in the seasons and locations in which there was higher organic matter content (*R*^*2*^ = 0.74, *p* < 0.0001). This connection implies that Hg was transported and stored in the organic matter fraction of the sediment in the Buenaventura Bay estuary. Other studies have reported the influence of sedimentary organic matter content on total Hg content (Chakraborty et al. 2015; Sunderland et al. 2006).

However, Hg content in the sedimentary compartment was explained by not only sedimentary organic matter but also clay percentage. In general, greater quantities of clay and, consequently, higher T-Hg occurred in sediments at seasons in which freshwater inflows increased and at sites that were influenced by river discharges. In the internal zones of estuaries where there is greater density of mangroves and fewer waves, a higher percentage of mud occurs in the sediment, it could make the retention of heavy metals, such as Hg, efficient (Azevedo et al. 2011).

Regarding physicochemical water parameters, sediment T-Hg was high for sites and seasons with low pH, salinity, transparency, and temperature values, as well as for deep samples. These physicochemical water characteristics indicate the influence of fluvial entrances, which can source clays and organic matter and, consequently, enhance the sedimentary T-Hg content. On the other hand, Cogua et al. (2012) reported a positive relation between the pH of the water and sediment T-Hg content in Cartagena Bay.

In general, sediment T-Hg in Buenaventura Bay was low for sites and seasons with lower values of both pH and salinity. However, at the EE location in September, in the outer zone of the bay, the highest sediment T-Hg value occurred: 0.14 ± 0.02 g.g^−1^. This occurrence can be explained by hydrodynamics and the chemical roles of pH and salinity. With increasing salinity, Hg can be released in ionic form, potentially forming Hg complexes in the water column. These complexes could precipitate and move toward the sediment surface layers (Kongchum et al. 2006; Wasserman et al. 2002). The EE station probably became a backwater, causing considerable organic matter to settle. Because EE was influenced by marine conditions, pH and salinity were enhanced, allowing T-Hg to be retained in the sediment.

In addition, this location corresponds to relative higher pH that occurred in the waters of marine conditions, translating to low H^+^ concentration. These ions competed with Hg^+^ and Hg^2+^ cations for the negatively charged sites on the surfaces of silt, clay, and organics within the sediment (Ravichandran 2004). Consequently, in the month of September at the EE zone, the interactions of the parameters described above, with relative higher pH and salinity, improve the retention of T-Hg in sedimentary organic matter, meaning less transfer of Hg from sediment to organisms.

It has been reported that in estuarine ecosystems, the upper trophic levels show higher Hg content in their tissues (Coelho et al. 2013). In the study area, it was reported that crustaceans of families Penaeidae and Portunidae maintained feeding habits that categorized them as scavengers and opportunists (Baltazar 1997). With respect to the species *Callinectes arcuatus*, it has been reported that 21.4% of its diet is made up of shrimp and other crustaceans and that it is a voracious predator (Pineda and Madrid 1993). Perhaps for these reasons, mature individuals of *C. arcuatus*, *C. toxotes,* and *Menippe frontalis* were the species with the highest muscle T-Hg concentrations, exceeding 0.2 μg·g^−1^, which is the maximum level suggested for consumption by this vulnerable human population. Some mature individuals of *C. arcuatus* exceeded the concentration of 0.5 μg·g^−1^, which is the maximum level suggested for consumption by the general population (WHO 1990). The vulnerable population in this case refers to pregnant women, children, and the community that feeds from this source of protein on a daily basis; the general population is the population that consumes it sporadically. Other studies of Hg in the macroinvertebrates of Colombia’s coastal ecosystems have reported that 50% of crustaceans exceed the value of 0.5 μg·g^−1^ T-Hg (Olivero-Verbel et al. 2008) and that macroinvertebrates inhabiting deeper marine habitats do not reach such high Hg levels (Valdelamar et al. 2014; Velásquez and Cortés 1997).

Furthermore, Olivero-Verbel et al. (2008) noted that for the bay of Cartagena, there is a significant correlation (*p* < 0.0001, R^2^ = 0.349, n = 153) between organismal weight and muscle T-Hg content for the species *Callinectes sapidus* and *C. bocourti*. These authors also reported significant geographic differences in macroinvertebrate T-Hg content, probably due to the influence of geography with respect to the Hg source. During the present study in Buenaventura Bay, crabs of the species *C. arcuatus* showed the highest size-standardized muscle T-Hg concentration in November and at the IE station, in both cases ostensibly related to reduced salinity. The same pattern was found for the subspecies *S. aculeata aculeata*. In November and at IE, conditions of lower salinity predominated, while sediment T-Hg concentrations became elevated. This pattern emerges despite the nonfacilitation of sedimentary Hg sequestration by reduced salinity conditions (Wasserman et al. 2002). In summary, in the seasons and locations at which conditions of lower salinity occurred, Hg, in its biogeochemical cycle, could take alternative routes to the sediment, which resulted in higher Hg levels in the organisms.

### Bioaccumulation

The results of the bioaccumulation factor analysis show that the transfer of total Hg from sediments to organisms was more efficient in the seasons and locations with conditions closer to marine conditions. This pattern held despite the higher T-Hg levels in crab muscle under conditions of lower salinity, during which sediments contained more organic matter and Hg. Other studies have determined that the greater the sedimentary organic matter content, the lower is the bioaccumulation factor (Chen et al. 2009). In that case, the most efficient transfer of total Hg from sediments to organisms in marine conditions can be explained by changes in trophic habits on one hand and by contaminant kinetics on the other.

Regarding trophic habits, it has been reported that the Hg content in animal muscle is not only due to the entry of metal from sediments but also trophic relations and biomagnification (Oh et al. 2010; Olivero-Verbel et al. 2002). When marine conditions predominated in the Buenaventura Bay, greater diversity and abundance of macroinvertebrates occurred; this situation indicates switches or additions of trophic relations (Gamboa-García et al. 2018). Thus, under marine conditions of the estuary, the species *C. arcuatus* may have experienced seasonal changes in its trophic habits, which made the transfer of Hg to its tissues more efficient.

On the other hand, an inverse relation has been reported between bioaccumulation factors and Hg environmental concentrations. This relation can be explained by mechanisms such as active regulation, natural ontogenic concentrations, and chemical kinetics at elevated concentrations (DeForest et al. 2007). Concerning the chemical kinetics mechanism, the inverse relation between the bioaccumulation factor in crab muscle and sediment T-Hg content could be a result of organismal exposure to high T-Hg concentrations in conditions of lower salinity. Such a situation could generate saturation, whereas in marine conditions, a passive transport mechanism could have operated.

The present investigation’s strong correlations between organic matter and sediment T-Hg concentrations are similar to those reported along the southern New England coast by Taylor and Calabrese (2018). Furthermore, these same authors also reported a negative slope in the relation of sediments T-Hg to organismal T-Hg. It may be that in both southern New England and Buenaventura Bay, sedimentary organic matter is capturing Hg and, in consequence, reducing its bioavailability.
